# Decline in Drug Overdose Deaths After State Policy Changes — Florida, 2010–2012

**Published:** 2014-07-04

**Authors:** Hal Johnson, Leonard Paulozzi, Christina Porucznik, Karin Mack, Blake Herter

**Affiliations:** 1 Hal Johnson Consulting and Division of Disease Control and Health Promotion, Florida Department of Health; 2 Division of Unintentional Injury Prevention, National Center for Injury Prevention and Control, CDC; 3 Department of Family and Preventive Medicine, University of Utah; 4 Division of Analysis, Research, and Practice Integration, National Center for Injury Prevention and Control, CDC; 5 Bureau of Emergency Medical Oversight, Florida Department of Health

During 2003–2009, the number of deaths caused by drug overdose in Florida increased 61.0%, from 1,804 to 2,905, with especially large increases in deaths caused by the opioid pain reliever oxycodone and the benzodiazepine alprazolam (1). In response, Florida implemented various laws and enforcement actions as part of a comprehensive effort to reverse the trend. This report describes changes in overdose deaths for prescription and illicit drugs and changes in the prescribing of drugs frequently associated with these deaths in Florida after these policy changes. During 2010–2012, the number of drug overdose deaths decreased 16.7%, from 3,201 to 2,666, and the deaths per 100,000 persons decreased 17.7%, from 17.0 to 14.0. Death rates for prescription drugs overall decreased 23.2%, from 14.5 to 11.1 per 100,000 persons. The decline in the overdose deaths from oxycodone (52.1%) exceeded the decline for other opioid pain relievers, and the decline in deaths for alprazolam (35.6%) exceeded the decline for other benzodiazepines. Similar declines occurred in prescribing rates for these drugs during this period. The temporal association between the legislative and enforcement actions and the substantial declines in prescribing and overdose deaths, especially for drugs favored by pain clinics, suggests that the initiatives in Florida reduced prescription drug overdose fatalities.

Florida gained notoriety after 2007 because of the proliferation of pain clinics in the state that were prescribing large quantities of drugs for pain with little medical justification and were being used primarily by persons abusing or diverting opioid analgesics, benzodiazepines, and muscle relaxants (2). In 2010, Florida was also home to 98 of the 100 U. S. physicians who dispensed the highest quantities of oxycodone directly from their offices. In response, Florida enacted several measures to address prescribing that was inconsistent with best practices. The Florida legislature required that pain clinics treating pain with controlled substances register with the state by January 4, 2010. In February 2010, the Drug Enforcement Administration and various Florida law enforcement agencies began to work together in Operation Pill Nation (3). Pain clinic regulations were further expanded later in 2010. In February 2011, law enforcement conducted statewide raids, resulting in numerous arrests, seizures of assets, and pain clinic closures. In July of that year, coinciding with a public health emergency declaration by the Florida Surgeon General, the state legislature prohibited physician dispensing of schedule II or III drugs from their offices and activated regional strike forces to address the emergency. Mandatory dispenser reporting to the newly established prescription drug monitoring program began in September 2011. Finally, in 2012, the legislature expanded regulation of wholesale drug distributors and created the Statewide Task Force on Prescription Drug Abuse and Newborns.

Florida Medical Examiners Commission (FMEC) data from the period 2003–2012 were analyzed for this report. Florida has a regional system of 24 district medical examiners with jurisdiction over all drug-related deaths occurring in the state. Florida has established a unique system that requires each medical examiner to submit a report to the FMEC on every death in which a drug is detected in a decedent. The report includes information on the manner of death (unintentional, suicide, homicide, or undetermined) and which of 50 monitored drugs were detected in the decedent (including prescription drugs, illicit drugs, and alcohol). For each drug detected, the medical examiner determines whether it played a causal role in the death or was merely present (4). Only those deaths caused by one or more drugs (i.e., overdoses) were included in this analysis. Deaths were not restricted to Florida residents.

Drug overdose death rates per 100,000 Florida residents were computed using population estimates compiled by the Florida Department of Health in consultation with the Florida Legislature’s Office of Economic and Demographic Research.* Rates were calculated for deaths caused by all drugs, all prescription drugs, opioid analgesics (including oxycodone, methadone, hydrocodone, morphine, and hydromorphone), benzodiazepines (including alprazolam), carisoprodol (a muscle relaxant), illicit drugs (including heroin and cocaine), and alcohol. Most deaths included more than one drug, so rates (including those for alcohol) refer to deaths involving a drug type irrespective of whether they were single or multidrug overdoses. The statistical significance of changes in death rates from 2010 to 2012 was assessed using z-tests.

Rates of prescribing selected prescription drugs in Florida were calculated from statewide estimates of prescription counts from the IMS Health National Prescription Audit (NPA). NPA provides state level estimates of the numbers of prescriptions filled during 2008–2012. NPA estimates are based on a sample of approximately 57,000 pharmacies, which fill nearly 80% of the retail prescriptions in the United States. Confidence limits for the estimates are not available. All prescriptions, including refills, dispensed at retail pharmacies were included (5). Prescriptions were not restricted to those for Florida residents.

The rate of drug overdose deaths increased 58.9% during 2003–2010. The number of drug overdose deaths decreased 16.7%, from 3,201 to 2,666, and the rate decreased 17.7% during 2010 and 2012 (Table 1, Figure 1). This change was largely attributable to the decrease in prescription drug-related deaths, which peaked at 2,722 in 2010 and decreased to 2,116 in 2012. The prescription drug overdose death rate decreased 23.2% to 11.1 per 100,000 persons, the lowest rate since 2007. Opioid analgesic overdose deaths declined from 2,560 to 1,892, with a corresponding rate decrease of 27.0%. Oxycodone, methadone, and hydrocodone rates decreased, whereas morphine and hydromorphone rates increased. Benzodiazepine overdose death rates decreased 28.4%, with alprazolam rates down 35.6%. The rate of carisoprodol-related deaths also declined, but not significantly. Prescribing declined for drugs whose overdose rate declined and increased for drugs whose overdose rate increased. For example, oxycodone prescribing declined 24.0%, whereas morphine prescribing increased 37.6%. Overall illicit drug overdose death rates did not change significantly, although heroin overdose deaths increased from 48 to 108, a change from 0.3 to 0.6 per 100,000 persons. Alcohol overdose death rates were unchanged. The semiannual time trends in overdose rates for specific drugs indicate a steady decline beginning in 2011 rather than an abrupt decline following any one of the legislative and enforcement actions taken in Florida (Figure 2).

Although the oxycodone overdose death rate decreased across all demographic groups, the greatest declines were among males (57.0%) and non-Hispanic whites (52.6%) (Table 2). Decedents who were aged 0–24 years (67.0%) and 25–34 years (66.7%) showed larger decreases than older decedents. The rate of deaths ruled unintentional showed a larger decrease (53.9%) than those of suicide (37.8%) or undetermined intent (29.0%). Additionally, the rate of deaths in which oxycodone and alprazolam were both identified as causal declined 61.5%.

## Discussion

This analysis showed that policy changes in Florida were followed by declines in the prescribing of drugs, especially those favored by Florida prescribing dispensers and pain clinics, as well as by declines in overdose deaths involving those drugs. Florida has reported that approximately 250 pain clinics were closed by 2013, and the number of high-volume oxycodone dispensing prescribers declined from 98 in 2010 to 13 in 2012 and zero in 2013 (2). Law enforcement agencies in Florida also reported that rates of drug diversion (i.e., channeling of prescription drugs to illicit markets) declined during 2010–2012 (6). Preliminary data for the first half of 2013 from the FMEC indicate a continued decline in oxycodone and alprazolam overdose deaths (4). These changes might represent the first documented substantial decline in drug overdose mortality in any state during the past 10 years.

What is already known on this topic?

From 2003 to 2009, the number of deaths caused by drug overdose in Florida increased 61.0%, from 1,804 to 2,905. In 2010, Florida’s legislature implemented laws regulating pain clinics, and in 2011, prohibited prescribers from dispensing opioid analgesics from their offices.

What is added by this report?

After the implementation of legislation, overdose death rates for opioid analgesics declined 27.0%, from 13.6 to 9.9 per 100,000 persons, and overdose death rates for benzodiazepines declined 28.4%, from 6.9 to 5.0 per 100,000 persons. Heroin overdose death rates increased 122.4%, from 0.3 to 0.6 per 100,000, but the overall drug overdose death rate declined 17.7%, from 17.0 to 14.0 per 100,000.

What are the implications for public health practice?

State legislation that establishes oversight over pain management clinics or describes specific registration, licensure, or ownership requirements for such clinics, coupled with restrictions on dispensing controlled substances by prescribers, are promising interventions to limit prescription drug overdose deaths.

Although the combined state initiatives were followed by the desired effect, determining the extent of each policy’s contribution to the decline in overdose deaths in Florida is not possible. Declines in overdoses of oxycodone might also have been related to the transition in late 2010 to a formulation of extended-release oxycodone designed to be abuse-resistant (7), but most of the decline in oxycodone prescribing and overdoses occurred after 2011. The increase in deaths associated with heroin and hydromorphone and morphine after 2010 might be a sign of a switch to use of alternative opioids. However, the effect of such a switch was limited: 668 fewer opioid analgesic overdose deaths occurred in 2012, compared with 60 more heroin deaths. Heroin deaths fluctuated widely during 2003–2012, so other factors might be involved. Moreover, other states that did not experience declines in prescription opioid deaths have reported increases in heroin overdose deaths during 2010–2012 (8). National data indicate a substantial increase in heroin overdose deaths during 2010–2011 (CDC WONDER, unpublished data, 2014).

The findings in this report are subject to at least five limitations. First, rates might be overestimated by the inclusion of nonstate residents, but the impact of this factor on trends is likely to be small (Florida Medical Examiners Commission, unpublished data, 2005–2008). Second, deaths from heroin might be underestimated because only the metabolites of heroin, such as morphine, are usually present in postmortem toxicology specimens. For prescription drug overdose deaths, however, the FMEC data provide a more complete accounting than death certificates (9). Third, prescription counts are estimated by a proprietary method and therefore include an undisclosed amount of error. Fourth, the role of other factors that might have affected prescribing and/or overdose death rates during this period (e.g., greater awareness of the problem) could not be evaluated. The absence of similar recent drug-specific overdose mortality data from other states precluded a comparison with other jurisdictions not making policy changes. Finally, the data sources available for this investigation did not permit any assessment of potential unintended consequences of these policy changes, such as reduction of access to pain medication for legitimate prescribing indications.

Some of the measures introduced in Florida have been adopted by other states. For example, the number of states with pain clinic laws increased from three in 2010 to 11 in 2013 (10). However, more rigorous evaluations of such interventions using comparison populations are necessary. At present, state legislation that establishes oversight over pain management clinics or describes specific registration, licensure, or ownership requirements for such clinics, coupled with restrictions on dispensing controlled substances by prescribers, can be considered promising interventions to reduce prescription drug overdose deaths.

## Figures and Tables

**FIGURE 1:**
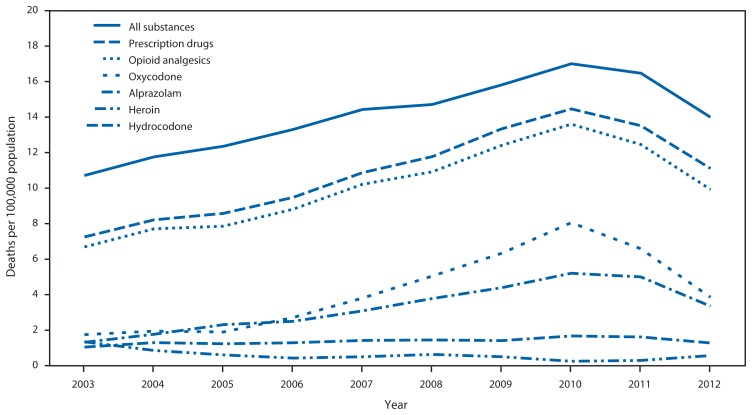
Overdose death rates^*^ for selected substances, by year — Florida, 2003–2012^†^ ^*^ Per 100,000 population. Based on Florida Department of Health resident population estimates, available at http://www.floridacharts.com/flquery/population/populationrpt.aspx. ^†^ The source of overdose death data is the Florida Medical Examiners Commission.

**FIGURE 2:**
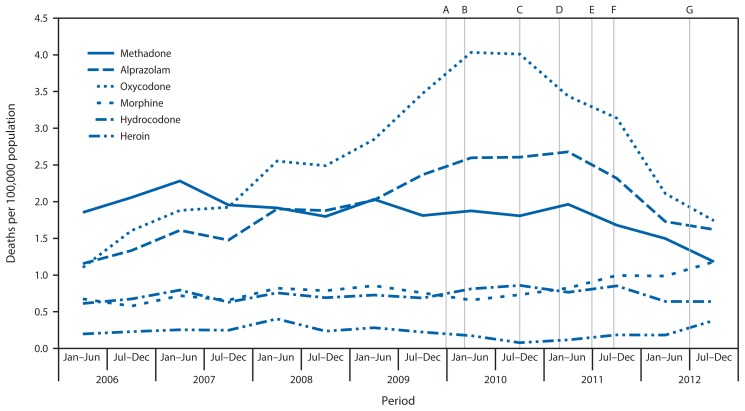
Semiannual drug overdose death rates^*^ for selected drugs, and selected prescription drug diversion and misuse actions taken — Florida, 2006–2012^†^ ^*^ Per 100,000 population. Based on Florida Department of Health resident population estimates, available at http://www.floridacharts.com/flquery/population/populationrpt.aspx. ^†^ The source of overdose death data is the Florida Medical Examiners Commission. A. January 4, 2010. Pain clinics must register. B. February, 2010. Operation Pill Nation: U.S. Drug Enforcement Agency and state and local law enforcement begin investigation of pain clinics. C. October 1, 2010. Pain clinic regulation expanded. D. February 23, 2011. Operation Pill Nation: joint law enforcement raids begin. E. July 1, 2011. Physician dispensing prohibited and statewide regional strike forces activated. F. September 1, 2011. Mandatory reporting to prescription drug monitoring program begins. G. July 1, 2012. Wholesale distributor regulations expanded.

**TABLE 1:** Overdose death rates,* number of overdose deaths, and prescribing (Rx) rates† for selected substances, by year — Florida, 2003–2012

Substance	Year										% change 2010 to 2012

	2003	2004	2005	2006	2007	2008	2009	2010	2011	2012	
**Prescription drugs**	**7.3**	**8.2**	**8.6**	**9.5**	**10.9**	**11.8**	**13.3**	**14.5**	**13.5**	**11.1**	**−23.2** §
	**1,239**	**1,436**	**1,534**	**1,730**	**2,012**	**2,195**	**2,496**	**2,722**	**2,560**	**2,116**	**−22.3**
Opioid analgesics	6.7	7.7	7.9	8.8	10.2	10.9	12.4	13.6	12.5	9.9	−27.0§
	1,142	1,347	1,405	1,608	1,891	2,037	2,323	2,560	2,359	1,892	−26.1
Oxycodone	1.8	1.9	1.9	2.7	3.8	5.0	6.3	8.1	6.6	3.9	−52.1§
	299	340	340	496	705	941	1,185	1,516	1,247	735	−51.5
*Rx rate*	*—*	*—*	*—*	*—*	*—*	*21,571*	*23,195*	*26,049*	*24,456*	*19,790*	−*24.0*
Methadone	2.1	3.2	3.5	3.9	4.2	3.7	3.8	3.7	3.6	2.7	−27.2§
	367	556	620	716	785	693	720	694	691	511	−26.4
*Rx rate*	*—*	*—*	*—*	*—*	*—*	*1,674*	*1,802*	*1,950*	*1,986*	*1,760*	−*9.8*
Hydrocodone	1.1	1.3	1.2	1.3	1.4	1.4	1.4	1.7	1.6	1.3	−23.1§
	180	228	221	236	264	270	265	315	307	245	−22.2
*Rx rate*	*—*	*—*	*—*	*—*	*—*	*34,409*	*34,335*	*33,184*	*32,685*	*29,970*	−*9.7*
Morphine	1.3	1.2	1.4	1.3	1.4	1.6	1.6	1.4	1.8	2.2	56.2§
	217	216	247	229	255	300	302	262	345	414	58.0
*Rx rate*	*—*	*—*	*—*	*—*	*—*	*2,222*	*2,564*	*2,693*	*3,028*	*3,706*	*37.6*
Hydromorphone	0.1	0.1	0.1	0.2	0.2	0.2	0.3	0.3	0.5	0.9	189.9§
	12	20	24	31	36	41	64	60	99	176	193.3
*Rx rate*	*—*	*—*	*—*	*—*	*—*	*863*	*1,109*	*1,133*	*1,403*	*1,790*	*58.0*
Other opioid analgesics	1.6	1.5	1.4	1.4	1.4	1.7	1.5	2.1	2.2	2.0	−4.5
	276	268	257	249	267	313	288	386	411	373	−3.4
Benzodiazepines	2.2	2.6	3.2	3.5	4.0	5.0	5.9	6.9	6.8	5.0	−28.4§
	376	460	574	632	743	929	1,099	1,305	1,294	945	−27.6
Alprazolam	1.3	1.8	2.3	2.5	3.1	3.8	4.4	5.2	5.0	3.4	−35.6§
	226	310	414	456	572	705	822	981	947	639	−34.9
*Rx rate*	*—*	*—*	*—*	*—*	*—*	*21,319*	*22,503*	*23,681*	*23,114*	*21,041*	−*11.1*
Other benzodiazepines	1.1	1.1	1.2	1.3	1.4	1.8	2.2	2.4	3.0	2.3	−5.0
	192	198	222	235	258	328	406	459	565	441	−3.9
Carisoprodol	0.3	0.5	0.5	0.4	0.5	0.5	0.5	0.6	0.8	0.5	−19.0
	45	81	96	74	88	84	98	111	153	91	−18.0
*Rx rate*	*—*	*—*	*—*	*—*	*—*	*4,585*	*4,719*	*4,883*	*4,668*	*3,649*	−*25.3*
**Illicit drugs**	**4.3**	**4.4**	**4.9**	**5.1**	**5.1**	**4.1**	**3.4**	**3.6**	**3.9**	**3.8**	**5.5**
	**737**	**771**	**882**	**936**	**935**	**768**	**635**	**678**	**739**	**724**	**6.8**
Heroin	1.3	0.9	0.6	0.4	0.5	0.6	0.5	0.3	0.3	0.6	122.4§
	230	150	109	78	93	119	95	48	57	108	125.0
Cocaine	3.2	3.4	4.1	4.5	4.6	3.5	2.8	3.0	3.2	2.9	−3.1
	541	591	732	829	843	648	529	561	604	550	−2.0
**Ethanol (alcohol)**	**1.6**	**1.7**	**1.9**	**2.1**	**2.5**	**2.6**	**3.0**	**3.0**	**3.1**	**3.0**	−**0.8**
	**279**	**293**	**343**	**378**	**466**	**489**	**559**	**572**	**590**	**574**	**0.3**
**All substances** ¶	**10.7**	**11.8**	**12.4**	**13.3**	**14.4**	**14.7**	**15.8**	**17.0**	**16.5**	**14.0**	**−17.7** §
	**1,829**	**2,056**	**2,210**	**2,427**	**2,670**	**2,742**	**2,960**	**3,201**	**3,120**	**2,666**	**−16.7**

* Per 100,000 population, based on Florida Department of Health resident population estimates, available at http://www.floridacharts.com/flquery/population/populationrpt.aspx. The source of overdose death data is the Florida Medical Examiners Commission.

† Per 100,000 population, based on Florida Department of Health resident population estimates. The source of prescribing data is IMS Health’s National Prescription Audit.

§ Change in rate is statistically significant at p<0.001. Changes in prescribing rates were not tested.

¶ Many deaths had more than one drug contributing to the death; thus, the sum of the rates in each column exceeds the total death rate.

**TABLE 2:** Oxycodone overdose death rate* and number of deaths, by selected characteristics — Florida, 2010 and 2012†

Characteristic	2010		2012		% change in rate
	
	Rate	No.	Rate	No.	
**Sex**					
Female	5.1	487	2.9	287	−41.8
Male	11.2	1029	4.8	448	−57.0
**Age group (yrs)**					
0–24	2.7	156	0.9	52	−67.0
25–34	17.3	394	5.8	136	−66.7
35–44	14.4	349	6.4	151	−55.7
45–54	15.0	412	8.4	225	−44.3
≥55	3.6	205	2.9	171	−19.2
**Race/Ethnicity**					
White, non-Hispanic	13.2	1446	6.3	683	−52.6
Black/Other, non-Hispanic	1.3	46	1.0	37	−21.4
Hispanic	0.6	24	0.3	15	−39.8
**Manner of death**					
Unintentional	7.2	1347	3.3	628	−53.9
Suicide	0.7	124	0.4	78	−37.8
Undetermined	0.2	39	0.1	28	−29.0
**Oxycodone and alprazolam**	3.3	627	1.3	244	−61.5
**Total**	**8.1**	**1516**	**3.9**	**735**	**−52.1**

* Per 100,000 population. Based on Florida Department of Health resident population estimates, available at http://www.floridacharts.com/flquery/population/populationrpt.aspx.

† The source of overdose death data is the Florida Medical Examiners Commission.

## Footnotes

* Data available at http://www.floridacharts.com/flquery/population/populationrpt.aspx.
